# Modern Sociology

**Published:** 1900-05-19

**Authors:** 


### Modern Sociology.

### THE TRAINING OF THE FEEBLE-MINDED.

An interesting paper was read to the members of the Childhood Society on March 13th by Dr. G. E. Shuttleworth, medical examiner of defective children for the London School Board, and formerly medical superintendent of the Royal Albert Asylum, Lancaster, on the "Training of Defective Children under the School Board." It is very desirable that this subject should receive the attention of the public, as many of the accusations brought against Board school education are really true only of its effect, or lack of effect, on children of less than average capacity, though they are often uttered as if the attempt to educate the average child was entirely a failure, or nearly so.

It was in Germany that the fact was first perceived that in all communities there are some children who are so constituted that they cannot profit by ordinary educational facilities. A committee of the Education Department which inquired into the subject reckons the proportion at 1 per cent. of the total school population. Observers in other countries seem to think that the percentage is higher than this. Mr. W. S. Monroe, of the Stanford University, California, came to the conclusion, after making investigations of over 10,000 children, that the proportion was nearer 3 per cent.; and Dr. E. A. Osborne, the superintendent of the California Home for the Training and Care of Feeble-minded Children, put the proportion of mentally deficient children as high as 10 per cent. of the population of school age. But whether the proportion be small or large, it is evident that there are everywhere to be found children who learn nothing at ordinary schools, who are often punished for a stupidity which they cannot help, and who by their presence and the demands they make on the teacher hinder the progress of the rest of the class. For such children special classes were established in Germany in 1863 and in Scandinavia more than twenty years ago. The London School Board formally approved of the institution of similar classes in 1891, but the first step was actually taken by the School Board of Leicester in 1892. The Boards of many other towns have since followed the example thus set, while others are preparing to do the same. In London there are now upwards of 50 special centres for the education of these feeble-minded children, the pupils at these numbering 2,125.

The decision as to what constitutes feeble-mindedness must come from a medical expert. Some teachers might be inclined to get rid of all their apparently dull pupils as requiring special treatment, when the real cause might be simple neglect or laziness. With the class from whom the larger number of Board school children are drawn these factors must always be reckoned on. Nor, though it is allied to it, must feeble-mindedness be confused with idiocy. The degree of mental infirmity must not be so great as to preclude the hope of improvement through special education, though varying degrees of dulness will be found. In suitable cases some children, after a course of special training, have, as Mrs. Burgwin reports in London, been able to return to the ordinary school standards, while a larger number, though not able to compete with their fellows in the ordinary schools, have been so far improved as to be able to follow useful employments. To show what special training may do for a child who seems hopelessly stupid, we may quote a case given by Miss Margaret Bancroft, of Haddonfield, New Jersey:—

"M. R. came to us last May (1893), stunted in growth, very deaf, very bad temper, no knowledge of letters or figures, could not thread a needle, nor do any kind of hand-work. She had been taught privately and in public schools, with the opinion of many that it was impossible to teach her to read, write, or sew."

This is the report a year later:—

"May, 1894. M. R. can read about half through her primer, all small words in her Bible, and in simple magazines, such as *Babyland;* can write spelling by dictation words of three and four letters. Has made a good attempt to write a short letter. Can do addition, simple subtraction without borrowing, understands the multiplication table in twos and threes, and has a good idea of the first lessons in geography and history, and simple object lessons. Excels in the gymnasium and in dancing. Can now sew, hem, outline, and hemstitch on coarse linen. Plain knitting and first pattern in lace making M. R. can do almost entirely alone, having a very little help from the teacher. She has commenced free hand drawing from the object, wood-work, and clay modelling; in all these branches she is making good progress. It was noticeable at first that she would tire after a few minutes when she was engaged in any manual work, but now she can work quite a long time without any apparent exhaustion. Her disposition is much improved."

This would be a good record even for a normal child. The moral of the record lies, perhaps, in the last sentence of it: "This work has been personal, hand to hand work, and we feel that if she had been in a class of many she would have remained a pupil unable to read, write, or do manual work." This is the one essential — individual, sympathetic training. Dr. Shuttleworth says that the more teaching can be individualised the better; the classes should never exceed 20 pupils to one teacher, and 10 would be better. The education of feeble-minded children is, of course, a work for which teachers would need to be specially trained; the ordinary teacher, however good in an ordinary school, would be of little use here. For this a training college is a desideratum. The needs of the pupils must be noted and considered. With the feeble minded, manual training often is effectual in developing intelligence when mere routine teaching of the elementary subjects was ineffectual. And thus trained, the feeble-minded child may become a useful citizen. In the Pennsylvanian Training School for Feeble-Minded Children the baking for the establishment is entrusted to six of the elder boys, who, according to the steward's report, "each week regularly turn 28 barrels of flour into good sweet bread, cakes, pies, &c." Under proper supervision, the feeble minded may do as good work as any other. But it must be remembered that the supervision is necessary. There is often moral weakness as well as mental, and left to themselves, the feeble-minded are apt to lapse into vice and crime. They will need guidance all their days.

